# Walking for transportation in Hong Kong Chinese urban elders: a cross-sectional study on what destinations matter and when

**DOI:** 10.1186/1479-5868-10-78

**Published:** 2013-06-20

**Authors:** Ester Cerin, Ka-yiu Lee, Anthony Barnett, Cindy HP Sit, Man-chin Cheung, Wai-man Chan, Janice M Johnston

**Affiliations:** 1 School of Exercise and Nutrition Sciences, Deakin University, 221 Burwood Highway, Burwood, Victoria, Australia; 2 Institute of Human Performance, The University of Hong Kong, Pokfulam Road, Hong Kong, SAR, Hong Kong; 3 School of Nursing, The University of Hong Kong, Sassoon Road, Hong Kong, SAR, Hong Kong; 4 Department of Sports Science and Physical Education, The Chinese University of Hong Kong, Shatin, N.T, Hong Kong, SAR, Hong Kong; 5 Department of Health, Elderly Health Service, 35/F Hopewell Centre, 183 Queens Road West, Wan Chai, Hong Kong, SAR, Hong Kong; 6 School of Public Health, University of Hong Kong, Sassoon Road, Hong Kong, SAR, Hong Kong

**Keywords:** Utilitarian walking, Elders, China, Environmental audit, Neighborhood, Destinations

## Abstract

**Background:**

Walking for transport can contribute to the accrual of health-enhancing levels of physical activity in elders. Identifying destinations and environmental conditions that facilitate this type of walking has public health significance. However, most findings are limited to Western, low-density locations, while a larger proportion of the global population resides in ultra-dense Asian metropolises. We investigated relationships of within-neighborhood objectively-measured destination categories and environmental attributes with walking for transport in 484 elders from an ultra-dense metropolis (Hong Kong).

**Methods:**

We estimated relationships of diversity (number of different types) and prevalence of within-neighborhood destination categories (environmental audits of 400 m buffers surrounding residential addresses) with transport-related walking (interviewer–administered questionnaire) in 484 Chinese-speaking elders able to walk unassisted and living in 32 neighborhoods stratified by socio-economic status and transport-related walkability. We examined the moderating effects of safety and pedestrian infrastructure-related neighborhood attributes on destination-walking associations.

**Results:**

Participants reported on average 569 and 254 min/week of overall and within-neighborhood walking for transport, respectively. The prevalence of public transit points and diversity of recreational destinations were positively related to overall walking for transport. The presence of a health clinic/service and place of worship, higher diversity in recreational destinations, and greater prevalence of non-food retails and services, food/grocery stores, and restaurants in the neighborhood were predictive of more within-neighborhood walking for transport. Neighborhood safety-related aspects moderated the relationship of overall walking for transport with the prevalence of public transit points, this being positive only in safe locations. Similar moderating effects of safety-related attributes were observed for the relationships of within-neighborhood walking for transport with diversity of recreational and entertainment destinations. Pedestrian-infrastructure attributes acted as moderators of associations of within-neighborhood walking for transport with prevalence of commercial destination categories. Composite destinations indices consisting of destination categories related to the specific measures of walking were positively associated with walking for transport.

**Conclusions:**

The availability of both non-commercial and commercial destinations may promote within-neighborhood walking for transport, while recreational facilities and public transit points may facilitate overall walking for transport. However, destination-rich areas need to also provide adequate levels of personal safety and a physically-unchallenging pedestrian network.

## Background

A physically active lifestyle is a key contributor to healthy aging [1]. Transport-related walking can be a substantial source of physical activity in elders [2]. By definition, the ability to walk to and from places depends on the availability and accessibility of relevant destinations. It is, thus, important to identify destinations that can potentially contribute to increased levels of walking for transport in elders and, also, identify environmental conditions that facilitate or hinder walking to such destinations.

The evidence about the categories of destinations that may impact on elders’ walking for transport is scarce. This is especially the case for non-Western populations. A handful of studies reported positive associations with diversity of land uses, access to commercial services, and/or public transit points [3-6]. However, some of these studies were entirely based on self-reports, while others used very generic objective measures of access to services (e.g., urbanization level) providing insufficient information on the categories of destinations (e.g., recreational or retail) that may matter to elders.

Importantly, no studies have attempted to examine the distinct contributions of destination prevalence and diversity to elders’ walking for transport. Here, destination prevalence refers to the number of destinations of a specific type within an area (e.g., neighborhood). For example, the number of all public recreational facilities in a neighborhood would provide a neighborhood-level measure of recreational destination prevalence. Thus, a neighborhood with two parks and a swimming pool (and no other recreational destinations) would have a recreational destination prevalence score of three facilities. In contrast, destination diversity refers to the number of types of destinations within an area. For example, the above-described neighborhood would have a recreational destination diversity score of two (two types of recreational destinations: park and swimming pool).

In general, destination prevalence and diversity represent measures of access and availability of services and, hence, are hypothesized to facilitate walking for transport (Van Cauwenberg et al., 2011). Distinguishing between these two destination dimensions is important because they may associate with walking for transport in different ways, which would have practical implications for the planning of environmental interventions. For example, diversity of public recreational destinations may have stronger positive effects on walking than prevalence. This is because recreational destinations of the same type (e.g., swimming pools) provide relatively uniform services/activities, while diverse public recreational facilities provide more activity options that can match residents’ preferences. In contrast, commercial destinations and services of the same type (e.g., Western fast food restaurants) may greatly vary in the range and quality of activities/products they offer. Consequently, having a greater prevalence of the same type of commercial destinations likely expands the range of choices and, hence, their relevance to a larger proportion of residents.

Destination prevalence and diversity are not the only neighborhood attributes that may encourage walking for transport. Aspects of neighborhood safety and pedestrian infrastructure may determine destination visitations and, thus, their effects on walking for transport. Although, there is some evidence that aspects of neighborhood safety [7,8] and pedestrian infrastructure [7,9] may have an additive effect on elders’ walking for transport, it is unknown whether they also interact with destination availability. It is plausible to assume that the availability of destinations may more positively impact on the walking behavior of residents of safer neighborhoods with a better pedestrian infrastructure.

Additionally, most studies did not examine environmental correlates of overall and within-neighborhood walking for transport. To understand how destination availability, neighborhood safety, and pedestrian infrastructure impact walking for transport, it is important to consider where walking occurs. This is particularly relevant to ultra-dense urban environments, as the present study site, with a developed public transport network allowing residents to easily access destinations and services outside their neighborhoods and, thus, compensate for the lack of within-neighborhood facilities. In support of this contention, using data from this study sample, we found stronger associations of perceived land use mix with within-neighborhood than overall walking for transport [3]. In other words, it appears that Hong Kong elders that do not have access to a variety of facilities (high levels of land use mix) in their neighborhood, can easily access other destination-rich areas by public transport and then walk to/from destinations in these areas.

To address the research gaps outlined above, this study examined associations of objectively-measured prevalence and diversity of nine destination categories with overall and within-neighborhood walking for transport in Chinese elders residing in Hong Kong, an ultra-dense metropolis [10-12]. We also examined the moderating effects of neighborhood safety and pedestrian infrastructure aspects on the above associations.

It was hypothesized that (1) compared to overall walking for transport, within-neighborhood walking for transport would be more strongly positively associated with each destination measure, with the exception of public transit points, which may facilitate walking outside the neighborhood [3]; (2) more favorable levels of neighborhood safety and pedestrian infrastructure would strengthen the positive associations of destination measures with overall and within-neighborhood walking for transport; (3) diversity measures of non-commercial destinations providing relatively uniform services (e.g., government, health services, or public recreational facilities) would be more strongly related to walking than their respective prevalence measures, and the opposite would hold for commercial destinations (e.g., food stores).

## Methods

### Participants and procedure

Ethical clearance was obtained from the Department of Health Ethics Committee (Hong Kong Special Administrative Region) and the Human Research Ethics Committee for Non-Clinical Faculties of the University of Hong Kong. In 2007–2008, Hong Kong residents aged 65+ (42% males; 33% 75+ year old; 13% with no formal education; 48% with primary and 39% with at least secondary education) were recruited from membership lists of four out of 18 Hong Kong Elderly Health Centres (EHCs) representing catchment areas of low- and high- transport-related walkability stratified by socio-economic status (SES) (response rate: 78%). Area SES was defined using census data on median monthly household income and percentage of owner-occupiers. Transport-related walkability was operationalized using Geographic Information Systems (GIS) data on household, intersection, and commercial/service destination densities. All EHCs were first ranked by SES of their catchment areas and categorized into low and high SES based on median split. Each EHCs SES category was then ranked by transport-related walkability and the EHCs with the bottom and top scores on walkability were selected. This type of strategy ensured that the variability in built-environment exposure measures related to walking for transport was maximized [13,14], with actual commercial/service destination densities ranging from 72 to 1643 units/km^2^ in the selected EHC catchment areas [13]. The EHCs, consisting of ~38 500 members, were established by the Department of Health of the Hong Kong SAR with the aim of providing membership-based comprehensive primary care services to residents aged 65+ years. EHC’s members are generally representative of the Hong Kong population of Chinese elders [15].

Eight street blocks with at least 25 residing EHC members were randomly selected without replacement in each of four EHC catchment areas (total of 32 street blocks). Approximately 15 participants with no diagnosed cognitive impairment, and able to walk unassisted (based on medical assessments), were randomly selected and recruited from each of the 32 blocks via an invitation letter followed up by a phone call. After providing written informed consent, they took part in a 40-minute face-to-face interviewer-administered survey. Participants received grocery vouchers as participation incentives. Environmental audits of the neighborhoods of residence of the study participants were conducted during daylight (10am-6pm).

### Instruments

#### Socio-demographic characteristics

Data on gender, age, and educational attainment were obtained using a short socio-demographic questionnaire.

#### Walking for transport

Walking for transport was defined as walking undertaken for the purpose of going to/from places, which is to be distinguished from recreational walking. Last 7-day weekly minutes of walking for transport, irrespective of location, were estimated using the Chinese version of the International Physical Activity Questionnaire – Long Form (IPAQ-LC) [16]. The reliability and validity of the IPAQ-LC among Hong Kong elders mirrored those of international studies [17]. Weekly minutes of walking for transport within the neighborhood were estimated using relevant items of the Neighborhood Walking Questionnaire – Chinese version for Seniors (NWQ-CS) [18]. Participants reported the usual number of weekly minutes undertaken within an area up to 15-minute walk from home, a commonly-used operational definition of neighborhood [19,20]. The NWQ-CS measure used in this study showed moderate reliability and validity [18].

#### Environmental audits

Attributes of the neighborhood environment were gauged using the Environment in Asia Scan Tool – Hong Kong version (EAST-HK; [12]), a validated environmental audit tool that was developed for use in Hong Kong and other densely-populated Asian metropolises. For the purpose of this study, we used dichotomous items (Present/Absent) on categories of destinations hypothesized to be relevant to Hong Kong elders based on pilot data [13]: *public transit points* (1 item); *recreational* (5 items); *places of worship* (1 item); *health clinics* (1 item); *government/public* (e.g., community center, post office, schools; 5 items); *entertainment* (e.g., movie/theatre; Hong Kong Jockey Club betting branches; 3 items); *non-food retail and services* (e.g., bank, barber, clothing; 7 items); *food and grocery stores* (e.g., supermarket, fresh-food market; 4 items); and *restaurants* (e.g., Western non-fast food restaurant, Western fast food restaurant; 5 items). Items measuring *safety* [traffic safety (3 items); crime (4 items); presence of stray animals (1 item); street lights (1 item)] and *infrastructure* [path obstructions (e.g., roadwork, motor vehicles parked on footpaths, hawkers; 4 items); good path conditions (2 items); sloping street (1 item); and public services (presence of public toilets and sitting facilities; 2 items)] were also employed.

Trained auditors collected data on both sides of each street segment (1536 in total) within 400m road-network distance from the participants’ residential blocks (n=32). A street segment was defined as a section of a street between intersections. Auditors were instructed to record destinations visible from the street. In the case of multi-story, mixed-use buildings, they were required to consult the directory of services usually located on the ground or first floor of the building. The average data collection time per street segment was ~11 minutes. Inter-rater agreement was moderate to perfect [12]. The 400 m road-network distance was selected based on previous studies on elders [21], budgetary constraints (not permitting the conduct of larger-scale environmental audits), and estimates of average distance walked in Hong Kong elders in 10–15 minutes of perceived time. Specifically, pilot data on 20 participants indicated that they overestimated the time walked by ~25-30% (15 perceived minutes of walking equivalent to 10–11 actual minutes) and walked at a self-paced speed of 2.8 km/hr in urban environments. These data suggest that Hong Kong elders on average cover a distance of 400-500 m in 15 perceived minutes of walking.

### Data processing and analysis

#### Data processing of EAST-HK data

Environmental data were aggregated by residential block areas representing neighborhoods. In this study, *destination prevalence* for single-item destination categories (health centers/clinics; places of worship; and public transit points) was operationalized as the number of street segments within a neighborhood with at least one destination of a specific category. To obtain prevalence measures of multiple-item destination categories, the numbers of neighborhood street segments with specific types of facilities falling within a category (e.g., restaurants) were computed and summed up. For example, if a neighborhood had Western fast-food restaurants in five street segments, Chinese non-fast food restaurants in three street segments, and no other types of restaurants, it would be assigned a score of 8 on the prevalence measure for restaurants. We counted number of street segments with a destination rather than number of destinations per street segment because the latter option is not feasible in ultra-dense metropolises such as Hong Kong with extreme levels of commercial/service destination density exceeding 1600 units/km^2^[13].

*Destination diversity* for single-item destination categories was operationalized as a specific category being present or absent in a specific neighborhood. Diversity measures of multiple-item destination categories were each operationalized as the number of different types of facilities within the neighborhood. For example, given that data were collected on five different types of recreational facilities, diversity scores on this destination category could range from 0 (no recreational facilities) to 5 (all five different types of facilities present in the neighborhood).

Aggregated scores of *safety* and *infrastructure* attributes measured with a single item represented the percentage of street segments within a neighborhood with the specific attribute, while scores of safety and infrastructure attributes measured by multiple items represented the percentage of the highest obtainable score averaged across street segments falling within a neighborhood. For example, given that the highest obtainable score on ‘path obstructions’ was 4, an average score of 2.6 across street segments would correspond to 65% of the maximal score (i.e., a score of 65 out of 100).

### Data analysis

Descriptive statistics were computed for all variables. Generalized linear models (GLMs) with gamma variance and logarithmic link functions, accounting for neighborhood-level clustering effects, were used to examine associations of neighborhood environmental characteristics with walking for transport variables. The gamma function was chosen because the outcome variables were positively skewed, with standard deviation values similar to the respective means [22]. All models were adjusted for gender, educational attainment, age, and neighborhood-level SES. The results from the GLMs were expressed as antilogarithms of the regression coefficients (e^b^) and their 95% confidence intervals, which are to be interpreted as the proportional increase or decrease in mean outcome associated with a 1 unit increase in the predictor.

A first set of GLMs estimated the independent contribution of socio-demographic, environmental safety, and infrastructure covariates to the explanation of overall and within-neighborhood walking for transport. A second set of GLMs estimated the relationships of single destination prevalence and diversity variables with the two outcomes, adjusting for the above covariates. Separate models estimated two-way interaction effects of destination prevalence and diversity variables by safety and infrastructure-related environmental attributes.

Destination prevalence and diversity variables that yielded main and/or interaction effects significant at a 0.05 probability level were included in two composite destination indices, one for overall the other for within-neighborhood walking for transport. It was not possible to enter the original individual destination prevalence and diversity variables in a multiple-predictor model because some of them were relatively strongly correlated (average *r* = 0.47; range 0.15 to 0.96). As there were two variables for each category of destination (e.g., diversity and prevalence scores), the variable with the strongest association (as per their *p* value) with a specific outcome variable was selected for the respective destination index. Candidate variables were standardized (z-score) and summed up to obtain an index. The indices were used as predictors in final GLMs, which also included significant covariates and interaction terms. Continuous variables were centered around the mean. Significant interaction terms were probed by estimating associations at values of the moderating environmental attributes 1 standard deviation below and above their mean [23]. The association at the average value of the moderator corresponded to the main effect as variables were centered around their means. For moderators with a standard deviation greater than the mean, associations at the smallest possible value of the moderating attribute and at 1 standard deviation above its mean were estimated. All analyses were conducted using Stata 10.1.

## Results

### Descriptive statistics

Participants reported an average of 569 (SD = 452) weekly minutes of overall (median = 420; interquartile range=630), and 254 (SD = 262) weekly minutes of within-neighborhood walking for transport (median = 175; interquartile range = 290). All study neighborhoods had at least one public transit point and a recreational facility (Table 1). The level of diversity was the lowest for entertainment destinations and the highest for stores and restaurants (>72% of maximal value on diversity score). The presence of stray animals and signs of crime/disorder was low, while street lights and well-maintained sidewalks were common features in all selected neighborhoods.

**Table 1:** Descriptive statistics of neighborhood environmental attributes (32 neighborhoods)

**Environmental attributes (theoretical range)**	***M *( *SD *)**	**Min-Max**
*Destination prevalence*^*a*^		
Health clinics/services (0–77)	4.0 (4.1)	0-14
Places of worship (0–77)	1.7 (2.2)	0-8
Public transit points (0–77)	7.4 (4.5)	1-22
Recreational destinations (0–308)	9.4 (5.2)	1-19
Government/public facilities (0–385)	7.1 (5.6)	0-31
Entertainment destinations (0–231)	2.6 (2.8)	0-12
Non-food retail and services (0–539)	19.7 (21.0)	0-80
Food and grocery stores (0–308)	13.2 (10.7)	0-46
Restaurants (0–385)	19.4 (20.1)	0-82
*Destination diversity*^*b*^		
Health clinics/services (0–1)	0.8 (0.4)	0-1
Places of worship (0–1)	0.5 (0.5)	0-1
Public transit points (0–1)	1.0 (0.0)	1-1
Recreational destinations (0–5)	2.4 (0.9)	1-5
Government/public facilities (0–5)	2.6 (1.2)	0-5
Entertainment destinations (0–3)	1.1 (1.0)	0-3
Non-food retail and services (0–7)	5.1 (2.4)	0-7
Food and grocery stores (0–4)	3.3 (1.0)	0-4
Restaurants (0–5)	3.6 (1.2)	0-5
*Infrastructure*^*c*^		
Sloping street (0–100)	14 (22)	0-75
Public facilities (0–100)	16 (9)	0-36
Good path conditions (0–100)	90 (7)	62-100
Path obstructions (0–100)	17 (13)	0-48
*Safety*^*c*^		
Stray animals (0–100)	2 (5)	0-20
Street lights (0–100)	85 (12)	36-100
Signs of crime/disorder (0–100)	2 (4)	0-12
Pedestrian safety (0–100)	55 (16)	24-83

*Notes.*^a^ Destination prevalence measures for a specific destination category (e.g., restaurants) represent the sum of the number of neighborhood street segments with specific destination types (e.g., specific types of restaurants) falling within the destination category. ^b^ Destination diversity measures represent the number of different types of facilities falling in the same destination category found in a neighborhood. ^c^ Safety- and infrastructure-related measures represent the percentage of the highest obtainable scores averaged across street segments falling within a neighborhood.

### Associations of neighborhood safety and infrastructure characteristics with walking for transport

When adjusting for socio-demographic characteristics, signs of crime/disorder were positively associated with both overall (e^b^ = 1.22; 95% CI = 1.01, 1.47; *p *= .039) and within-neighborhood walking for transport (e^b^ = 1.28; 95% CI = 1.05, 1.57; *p *= .015). The prevalence of street lights was positively (e^b^ = 1.02; 95% CI = 1.01, 1.03; *p*<.001), while the prevalence of stray animals negatively (e^b^ = 0.97; 95% CI = 0.94, 1.00; *p *= .050), related to within-neighborhood walking for transport. No significant associations were found between the two walking outcomes and pedestrian safety, sloping streets, public facilities, path obstructions and good path conditions.

### Associations of single destination prevalence and diversity variables with walking for transport

#### Overall walking for transport

Only two types of destinations showed some positive significant associations with weekly minutes of overall walking for transport. These were the prevalence of public transit points and diversity of recreational destinations (e^b^ = 1.13; 95% CI = 1.02, 1.25; *p = *.017). The association with the first variable was moderated by the prevalence of stray animals. The number of street segments with public transit points (public transit points prevalence score) was related to overall walking for transport in residents of neighborhoods with no stray animals (e^b^ = 1.03; 95% CI = 1.01, 1.05; *p = *.006), but not in residents of neighborhoods with average (e^b^ = 1.01; 95% CI = 0.99, 1.03; *p = *.118) or above average prevalence of stray animals (e^b^ = 0.99; 95% CI = 0.96, 1.02; *p = *.398).

#### Within-neighborhood walking for transport

Table 2 shows the associations of prevalence and diversity measures of various destination categories with weekly minutes of within-neighborhood walking for transport. The presence (diversity score) of at least one health clinic/service and place for worship in the neighborhood were both predictive of 45% higher levels of within-neighborhood walking for transport (Table 2). A unit higher score on the diversity measure of recreational destinations was associated with 16% more weekly minutes of within-neighborhood walking for transport. This association was moderated by signs of crime/disorder and the presence of stray animals, whereby recreational destination diversity was associated with walking only at average and below average levels of the two moderators (Table 2). The association of entertainment destination diversity with within-neighborhood walking for transport was also moderated by signs of crime/disorder, whereby a positive association was observed only in the absence of signs of crime/disorder. The prevalence but not diversity measures of non-food-retail and services, food and grocery stores, and restaurants were positively related with within-neighborhood walking for transport. These associations were moderated by path obstructions and/or sloping street segments in the neighborhood. Significant positive associations were observed for non-food retail and services, and food/grocery stores only at average or below average levels of sloping streets. While the associations of food and grocery stores with within-neighborhood walking for transport were positive at average or below average levels of path obstructions, those with non-food retail and services were positive at average or above average levels of path obstructions (Table 2).

**Table 2:** Associations of destination prevalence and diversity measures with weekly minutes of within-neighborhood walking for transport (N = 484)

**Destination categories**	**Prevalence score**			**Diversity score**		
	**e**^**b**^	**95% CI**	***p***	**e**^**b**^	**95% CI**	***p***
***Main effects***						
Health clinics/ services	1.03	0.97, 1.09	.501	1.45^a^	1.06, 1.99	.048
Places of worship	1.06	0.95, 1.17	.463	1.45^a^	1.09, 1.92	.031
Public transit point	1.02	0.97, 1.08	.592	NA	NA	NA
Recreation	0.99	0.96, 1.03	.650	1.16^b^	1.04, 1.31	.008
Government/public	1.00	0.98, 1.03	.753	0.92	0.78, 1.09	.489
Entertainment	1.00	0.96, 1.05	.797	1.08	0.95, 1.23	.400
Non-food retail and services	1.01^a^	1.00, 1.01	.047	1.08	0.98, 1.20	.239
Food and grocery stores	1.02^a^	1.00, 1.03	.044	1.10	0.99, 1.23	.165
Restaurants	1.01^c^	1.01, 1.02	<.001	1.12	0.93, 1.35	.391
***Interaction effects***						
*Destination category by Environmental attribute*						
Recreation by Signs of crime/disorder						
Association at No signs of crime/disorder	-	-		1.19^b^	1.06, 1.33	.010
Association at 1SD above average Signs of crime/disorder	-	-		0.84	0.62, 1.13	.424
Recreation by Stray animals						
Association at No stray animals	-	-		1.23^b^	1.06, 1.45	.009
Association at 1SD above average Stray animals	-	-		0.71	0.49, 1.05	.155
Entertainment by Signs of crime/disorder						
Association at No signs of crime/disorder	-	-		1.35^b^	1.08, 1.71	.002
Association at 1SD above average Signs of crime/disorder	-	-		0.77	0.59, 1.02	.125
Non-food retail and services by Path obstructions						
Association at 1SD below average Path obstructions	1.00	0.99, 1.01	.798	-	-	
Association at 1SD above average Path obstructions	1.01^b^	1.00, 1.03	.008	-	-	
Non-food retail and services by Sloping streets						
Association at No sloping streets	1.01^b^	1.00, 1.02	.010	-	-	
Association at 1SD above average Sloping streets	1.00	1.00, 1.01	.148	-	-	
Food and grocery stores by Path obstructions						
Association at 1SD below average Path obstructions	1.02^b^	1.01, 1.05	<.001	-	-	
Association at 1SD above average Path obstructions	1.00	0.99, 1.02	.668	-	-	
Food and grocery stores by Sloping streets						
Association at No sloping streets	1.02^a^	1.01, 1.04	.032	-	-	
Association at 1SD above average Sloping streets	1.01	0.99, 1.02	.471	-	-	

Note. *NA* not applicable. ^a^ p<.05; ^b^ p<.01; ^c^ p<.001; e^b^ = antilogarithm of regression coefficient; 95% *CI* 95% confidence intervals; *p* = *p*-value. The antilogarithm of the regression coefficient is interpreted as the proportional increase (if >1.00) or decrease (if <1.00) in the outcome associated with a 1 unit increase in the predictor. All models adjusted for age, gender, educational attainment, neighborhood socio-economic status, neighborhood clustering effects, neighborhood safety, and infrastructure attributes.

### Associations of composite destination indices with walking for transport

#### Overall walking for transport

A destination index consisting of the sum of the z-scores of prevalence of public transit points and recreational destination diversity, the two destination variables related to this outcome (see above), was computed (M=0.0; SD=1.9; Min=−3.1; Max=6.2). The index was significantly positively related to overall walking for transport (e^b^ = 1.06; 95% CI = 1.02, 1.10; *p=*.002). No significant interaction effects with neighborhood safety or infrastructure characteristics were observed.

#### Within-neighborhood walking for transport

Another destination index consisting of the sum of the z-scores of prevalence measures of non-food retail and services, food and grocery stores, and restaurants, and diversity measures of entertainment destinations, recreational destinations, places of worship, and health clinics/services was computed (M=0.0; SD=4.7; Min = −7.3; Max=11.3). The index was significantly positively related to within-neighborhood walking for transport at average (e^b^ = 1.05; 95% CI = 1.03, 1.09; *p<*.001) and below average (e^b^ = 1.08; 95% CI = 1.06, 1.12; *p<*.001), but not at 1SD above average levels of path obstructions (e^b^ = 1.02; 95% CI = 1.00, 1.06; *p = *.103). No other significant interaction effects were observed with other safety and infrastructure variables.

## Discussion

We estimated associations of objectively-measured availability of nine different categories of destinations with overall and within-neighborhood walking for transport in a sample of elders living in an ultra-dense Chinese metropolis (Hong Kong). Importantly, this is the first study to examine moderating effects of safety and infrastructure on destination-walking relationships in any population of elders.

As noted previously [3], the level of self-reported transport-related walking among Hong Kong elders was strikingly higher than that observed in corresponding Western samples [5], but similar to that previously reported in Mainland Chinese elders [24]. The observed large volumes of walking for transport may be due to cultural differences [25], more walkable and destination-rich environments [26], as well as lower levels of car ownerships in the study site [3]. Although the levels of transport-related walking were generally high, significant destination-walking associations were observed, suggesting that environmental factors may be at least in part responsible for the high levels of walking.

### Overall walking for transport

As hypothesized, and mirroring the findings observed in relation to perceived access to destinations [3], destination-walking associations were stronger and more consistent for within-neighborhood than overall walking for transport. This suggests that the compact built form and well-developed and affordable public transportation network of Hong Kong may make it possible for residents of destination-poor neighborhoods to easily access more walkable, amenity-rich neighborhoods and accumulate significant amounts of walking outside their neighborhood. In line with our hypotheses, public transit point prevalence was significantly associated with overall but not with within-neighborhood walking for transport. The potential importance of public transport accessibility for the promotion of an active lifestyle in elders has been observed in other geographical locations with lower levels of automobile dependency, such as Belgium [6]. Yet, in the present study, the association of public transit points with overall walking for transport was only significant in residents of neighborhoods with no stray animals. The presence of stray dogs may be perceived as a threat to personal safety and, consequently, deter older residents from waiting for public transport at transit points.

Recreational destination diversity was the only other destination variable related to overall walking for transport. This suggests that the availability of a variety of recreational facilities in the neighborhood may encourage walking to, and visitation of, such facilities by Hong Kong elders, who, however, may not tend to compensate for the lack of recreational destinations in their neighborhood by visiting other areas. Similar findings have been observed in a sample of Japanese elders, whereby perceived access to exercise facilities in the neighborhood emerged as a significant predictor of overall walking for transport [4]. Active recreational pursuits represent discretionary activities that, unlike activities essential for daily living (e.g., eating and self-care), may not be undertaken if relevant facilities are inconveniently located (outside the neighborhood). Thus, easily accessible recreational facilities may represent a discretionary category of destinations that uniquely contributes to overall walking for transport in Hong Kong elders.

A composite destination index including both standardized measures of prevalence of public transit points and recreational destination diversity revealed that residents of areas with the highest score on the index would report 68% (95% CI: 21%, 133%) more overall walking for transport that their lowest score counterparts, which represents a substantial effect.

### Within-neighborhood walking for transport

All types of destinations with the exception of public transit points and government/public facilities showed some significant positive associations with within-neighborhood walking for transport. As hypothesized, prevalence (rather than diversity) measures of commercial destinations were superior predictors of within-neighborhood walking for transport, while only diversity measures of non-commercial destinations were significantly associated with this type of walking. These findings are important as they highlight how different ways of quantifying destination variables may yield contrasting results.

Some of the observed destination-walking associations were independent of neighborhood safety and pedestrian infrastructure. The presence of health clinics/services and places of worship, and the prevalence of restaurants were significantly positively associated with within-neighborhood walking for transport across all examined environmental conditions. This suggests that health care, participation in religious practices and dining out may be activities particularly important to Hong Kong elders. While it is not unusual for elders to have health-related concerns and engage in religious practices, it might seem odd that they would regularly dine out. In reality, Hong Kong Chinese elders typically meet up with friends or family for usually inexpensive, traditional Chinese-style morning and afternoon tea.

Only recreational destination diversity, which was mainly represented by outdoor facilities, showed a significant interaction with prevalence of stray animals. This is understandable as they can be a potential threat to pedestrians and users of such facilities. Signs of crime/disorder appeared to deter the use of various recreational destinations and entertainment facilities, while a challenging pedestrian environment (sloping streets and path obstructions) suppressed otherwise positive relationships of within-neighborhood walking with the prevalence of destinations typically associated with shopping and, thus, carrying loads (non-food retail, food and grocery stores). The likelihood of elders’ walking to specific services and facilities in the local neighborhood appears to depend on how easily and safely they can be reached.

However, this study suggests also that, in the absence of relevant destinations, a good pedestrian neighborhood infrastructure is not sufficient to promote utilitarian walking in Hong Kong elders, which is line with observations from Japan [4]. In contrast, aspects of neighborhood safety such as the presence of street lights, stray animals, and signs of crime/disorder were independently related to walking for transport, with the latter characteristic showing an unexpected positive relationship. Signs of crime/disorder may measure aspects of SES that are not captured by individual- and area-level income and educational attainment. Thus, given that walking for transport is more prevalent in low-SES groups [27], it is not surprising that crime measures are sometimes positively related to this type of walking [4,5].

A composite destination index including all destination categories that were significant correlates of within-neighborhood walking for transport showed that, compared to participants living in areas with the lowest score, those living in areas with the highest score reported 187% (95% CI: 37%, 500%) more weekly minutes of this type of walking, highlighting the potential importance of the availability of such destinations for the promotion of active transportation in Hong Kong elders.

### Limitations

Although providing previously undocumented information on destination categories and environmental conditions that may facilitate walking for transport in Hong Kong elders able to walk unassisted, this study has limitations. This includes the cross-sectional design, which cannot account for self-selection bias. However, the low levels of car ownership (*NB*: none of the study participants owned a car) and residential mobility of Hong Kong elders makes this source of validity threat less relevant. Using self-report measures to assess overall and within-neighborhood walking for transport is another limitation. However, we were unable to use Global Positioning System (GPS) monitors to track walking behavior due to frequent loss of GPS signals in high-density areas. Additionally, objective measures such as accelerometers cannot assess purpose of walking and differentiate walking for transport from recreational walking.

## Conclusions

In conclusion, the compact, high-density, destination-rich nature of Hong Kong, easy access to a well-developed public transport network, low levels of car ownership as well as cultural factors may be responsible for the much higher levels of walking for transport observed in Hong Kong elders as compared to their Western counterparts. This study suggests that access to a variety of non-commercial and commercial destinations in the neighborhood appears to encourage within-neighborhood walking for transport. However, in order to substantially impact on walking, destination-rich areas may also need to provide adequate levels of personal safety and a physically-unchallenging pedestrian network. It appears that high-density, compact and well-interconnected urban cities, such as Hong Kong, allow elders living in relatively destination-poor neighborhoods easy access to areas with relevant destinations where they can accumulate substantial amounts of walking outside the neighborhood. The development of safe, destination-rich neighborhoods may be a challenging, long-term, and sometimes idealistic goal. Initiatives and policies aimed at providing affordable public transport routes linking destination-poor with destination-rich neighborhoods may offer a more realistic way to promote utilitarian walking in elders living in less walkable communities.

## Competing interests

The authors declare that they have no competing interests.

## Authors’ contributions

EC coordinated the study, analyzed the data, conceptualized, and wrote all drafts of the manuscript. AB contributed to data entry, conceptualization of the manuscript, and write-up of the drafts. CPHS, WMC, and MCC contributed to the questionnaire translation and adaptation. MCC, KYL, WMC, and JMJ helped with the coordination of the study and data collection. All authors critically reviewed drafts and approved the final version of the manuscript.
